# Expanding the capabilities of MuGENT for large-scale genetic engineering of the fastest-replicating species, *Vibrio natriegens*

**DOI:** 10.1128/spectrum.03964-23

**Published:** 2024-04-26

**Authors:** Liz D. Glasgo, Katie L. Lukasiak, Erik R. Zinser

**Affiliations:** 1 Department of Microbiology, University of Tennessee, Knoxville, Tennessee, USA; University of Manitoba, Winnipeg, Manitoba, Canada

**Keywords:** *Vibrio natriegens*, MuGENT, genetic engineering, deletion

## Abstract

The fastest replicating bacterium *Vibrio natriegens* is a rising workhorse for molecular and biotechnological research with established tools for efficient genetic manipulation. Here, we expand on the capabilities of multiplex genome editing by natural transformation (MuGENT) by identifying a neutral insertion site and showing how two selectable markers can be swapped at this site for sequential rounds of natural transformation. Second, we demonstrated that MuGENT can be used for complementation by gene insertion at an ectopic chromosomal locus. Additionally, we developed a robust method to cure the competence plasmid required to induce natural transformation. Finally, we demonstrated the ability of MuGENT to create massive deletions; the 280 kb deletion created in this study is one of the largest artificial deletions constructed in a single round of targeted mutagenesis of a bacterium. These methods each advance the genetic potential of *V. natriegens* and collectively expand upon its utility as an emerging model organism for synthetic biology.

**IMPORTANCE:**

*Vibrio natriegens* is an emerging model organism for molecular and biotechnological applications. Its fast growth, metabolic versatility, and ease of genetic manipulation provide an ideal platform for synthetic biology. Here, we develop and apply novel methods that expand the genetic capabilities of the *V. natriegens* model system. Prior studies developed a method to manipulate multiple regions of the chromosome in a single step. Here, we provide new resources that diversify the utility of this method. We also provide a technique to remove the required genetic tools from the cell once the manipulation is performed, thus establishing “clean” derivative cells. Finally, we show the full extent of this technique’s capability by generating one of the largest chromosomal deletions reported in the literature. Collectively, these new tools will be beneficial broadly to the *Vibrio* community and specifically to the advancement of *V. natriegens* as a model system.

## INTRODUCTION

*Vibrio natriegens* was first isolated and described in the late 1950s and early 1960s when it was found to have an astonishing doubling time of <10 minutes (1–3). *V. natriegens* is Gram-negative, halophilic, and non-pathogenic and offers a broad range of metabolic capabilities (4, 5). However, it was not until roughly the past decade that it gained attention as a promising model organism for industrial applications such as heterologous protein synthesis and small molecule production (6–9). Traditional genetic methods for *V. natriegens*, though labor intensive (4, 5, 9, 10), have been utilized in initial efforts to create strains of *V. natriegens* with more efficient substrate production and higher biomass yield by eliminating non-essential, potentially destabilizing, and/or resource costly regions of the genome (5, 11).

The development of multiplex genome editing by natural transformation (MuGENT) offers an advancement in these applications as a fast and efficient technique for genetic manipulation (6). Briefly, MuGENT exploits the natural transformation of linear PCR products into *V. natriegens* to make genome edits at multiple loci in a single round of transformation. Development and optimization of MuGENT in other *Vibrio* species, *Vibrio cholerae* and *Vibrio parahaemolyticus*, have included the ability to cure the inducer plasmid (6, 12–14), an important step currently lacking in *V. natriegens*. With the development of MuGENT, the potential to rapidly reduce the genome of *V.natriegens* in search of creating a more efficient chassis for heterologous expression is more feasible than ever, and importantly with the availability of comparative genomics data (10), identification of potentially dispensable genes is also easily achievable.

MuGENT requires the inclusion of a selectable marker to identify successful transformants. Traditionally during MuGENT, non-selectable products designed to make the desired genome edit(s) are cotransformed with a selectable product that replaces the endonuclease gene, *dns*, with an antibiotic-resistance marker (6). This mutation does not impact growth in rich media; however, mutations that eliminate functional genes may not be neutral in all environments. Another approach is to directly replace a gene of interest with the selectable marker itself, rather than cotransforming two separate products. While this may work if only a single deletion is needed, it limits the user’s ability to create successive genome edits. Therefore, we sought to identify an alternative fitness-neutral insertion site in *V. natriegens’* genome that avoids replacing a coding sequence to preserve cells as close to wild type as possible.

Induction of natural transformation in *V. natriegens* is achieved by ectopic expression of the competence regulator gene, *tfoX*, cloned on the plasmid pMMB*tfoX* (6). Plasmid curing after genome editing is important for subsequent physiological studies of the transformants but has proven challenging for this system. One efficient way to cure cells is to counterselect against a plasmid-encoding gene, such as *sacB* from *Bacillus subtilis. sacB* encodes levansucrase, and when expressed in Gram-negative bacteria, it causes lethality in the presence of sucrose (12). Strains cured of the plasmid are enriched among the colonies growing on sucrose plates and can subsequently be verified by PCR screening for the plasmid. Notably, *sacB* has been shown to function in *V. natriegens* as a counter-selection marker for allelic exchange on the *V. natriegens* chromosome (9). Here, we demonstrate that a *sacB*-containing derivative of pMMB*tfoX* (pMMB*sacBtfoX*) developed for *V. parahaemolyticus* (12) can also be used for plasmid curing in *V. natriegens*.

Complementation is the gold standard method to confirm a mutation is responsible for a specific phenotype. While plasmid-based complementation is commonplace in genetic studies, we wanted to test the ability to complement directly on the chromosome. Phenotypic rescue from a single copy located elsewhere on the chromosome may be preferable to rescue from copies on a multicopy plasmid if gene dosage is an important consideration. Complementation from the chromosome offers the additional advantage of a stably integrated gene that does not require selective pressure for the maintenance of the carrier plasmid (e.g., antibiotic resistance). We show that deletion of *oxyR*, a regulator of antioxidant genes (15), creates sensitivity to hydrogen peroxide. We complement this phenotype by inserting *oxyR* at a distant location on the chromosome.

Prior use of MuGENT has targeted the creation of only small deletions, 50–500 bp (6, 14), and the possibility of creating large, multi-gene deletions was unexplored. Therefore, we wanted to apply MuGENT to test the capability of generating large-scale genome deletions. As a proof of concept for the methods we developed, we generated a 280 kb deletion of Chromosome 2, the largest known deletion created using MuGENT and one of the largest created for any bacterium. Subsequently, sucrose counterselection allowed us to cure the deletion mutant of the pMMB*sacBtfoX* plasmid required for transformation. Importantly, and in distinction from *V. parahaemolyticus*, we find that curing of pMMB*sacBtfoX* was dependent on drastic reduction, but not elimination, of salt in the sucrose selection medium. We show that despite the removal of 239 genes, the Δ280 kb strain is viable in complex medium and suffers only a small growth rate defect compared to wildtype (WT) growth. The methods reported here provide valuable genetic tools for *V. natriegens* and demonstrate the potential for quick and efficient engineering of *V. natriegens* genome.

## MATERIALS AND METHODS

### Bacterial strains and culturing conditions

*V. natriegens* ATCC 14048 and *V. natriegens* ATCC 14048 harboring competence plasmid pMMB*tfox*, generously provided by Dr. Ankur Dalia, were used as the parent strains in this study. All strains and plasmids used in this study are listed in Table S1. *V. natriegens* was routinely grown in LB3 [LB with 3% (wt/vol) NaCl], and antibiotics were added at 100 µg/mL erythromycin (erm), 250 µg/mL spectinomycin (spec), 250 µg/mL kanamycin (kan), and 150 µg/mL ampicillin (amp) when appropriate, unless otherwise stated. All reagents were purchased from Thermo Fisher unless otherwise indicated. As an extension of the initial development of the MuGENT system in *V. natriegens*, all cultures in this study were likewise grown at 30°C (6). All liquid cultures were grown in an orbital shaker unless otherwise noted.

### Generation of selectable markers and mutant constructs

Selectable markers and deletion mutant constructs were created with three- or two-piece SOE (splicing-by-overlap extension) PCR essentially as previously described (6, 16). For selectable markers and *oxyR* complementation, 3 kb upstream and 3 kb downstream regions flanking the location of insertion were amplified by F1/R1 and F2/R2 primers, respectively (Table S2), and *oxyR* or antibiotic resistance markers (erm^R^ or spec^R^) were amplified from genomic or plasmid sources, respectively (Table S2). R1 and F2 primers have 20–23 bp nucleotide sequences added to the 5’ ends that are homologous to sequences added to the primers used to amplify the desired insert. This allows the sequences to be “stitched” together in SOE PCR. For three-piece SOE PCR, purified PCR products were added in a 50 ng : 50 ng : 50 ng ratio as the template DNA. PCR was run for 10–15 cycles without primers, and then the F1/R2 primers were added, and PCR was run for an additional 25–30 cycles. Thermocycler conditions were as follows: (i) 98°C – 30 seconds, (ii) 98°C – 10 seconds, (iii) 60°C – 10 seconds, (iv) 72°C – 30 seconds/kb, (v) repeat steps 2–4 10–30×, (vi) 72°C – 5 minutes, and (vii) 4°C – hold. SOE PCR products were gel purified with QIAquick Gel Extraction Kit (Qiagen) and used in transformations. The *ΔoxyR* and Δ280 kb constructs were generated in a similar manner, amplifying the 3 kb upstream and downstream regions flanking the site of deletion. These two pieces were purified using the QIAquick PCR Purification Kit (Qiagen) and added together in a 50 ng : 50 ng ratio as the template DNA for SOE PCR and run as described above. After purification, this product was used in transformation. All primers are listed in Table S2. All PCRs were performed using Phusion Plus Polymerase (Thermo Scientific) according to the manufacturer’s protocols. Products were visualized on 1.5% agarose gels using Midori Green (Bulldog Bio).

### pMMB*sacBtfoX* conjugation

To incorporate the pMMB*sacBtfoX* plasmid into our WT strain of *V. natriegens*, the plasmid was first introduced into a diaminopimelic acid (DAP) auxotroph strain of *Escherichia coli* WM3064 (Table S1) (17) via heat shock transformation. Overnight *E. coli* WM3064 cells were diluted 1:1,000 and grown for 3 hours at 37°C to mid-log. Cells were pelleted and resuspended in 100 µL of 0.1 M CaCl_2_. Three microliters of plasmid was added to the cells, and the mixture was placed on ice for 5 minutes. Then, the mixture was heat shocked at 42°C for 1 minute. Cells were placed back on ice while 0.4 mL of lysogeny broth (LB) was added. Then, the cells were grown out for 30 minutes at 37°C in a roller drum. Cells were plated onto LB + 50 µg/mL kanamycin + 500 µM DAP. To conjugate the plasmid into *V. natriegens*, overnights of each strain were grown: *E. coli* WM3064-pMMB*sacBtfoX* was grown in LB supplemented with kanamycin and DAP at 37°C, and *V. natriegens* was grown in LB3 at 30°C. Cells were pelleted and resuspended three times in fresh LB. Cells were mixed together in various ratios of the donor to recipient 1:9, 9:1, 1:4, 4:1, 1:1, 0:1, and 1:0 in total volumes of 100 µL. The mixtures were spotted onto LB plates and incubated for 3–5 hours at 30°C. The cells were then resuspended from the plates with 1 mL of LB and plated onto LB3 + kanamycin plates lacking DAP (to eliminate the *E. coli* donor strain) and incubated at 30°C overnight. Colonies formed on plates for the 1:9, 9:1, and 1:1 dilutions, but not for the 1:4, 4:1, or the donor alone or recipient alone controls (1:0; 0:1).

### Natural transformation/MuGENT

Natural transformation was performed as previously described (6). Briefly, cells containing the competence plasmid pMMB*tfox* or pMMB*sacBtfoX* were grown overnight at 30°C, shaking (250 rpm) in LB3 supplemented with 100 µM isopropyl β-D-1-thiogalactopyranoside (IPTG) and appropriate antibiotic. Cells were then diluted 1:1,000 into 350 µL of Instant Ocean (28 g/L; Spectrum Brands) with 100 µM IPTG. For the natural transformation of selectable markers, 50 ng of PCR construct was added. For MuGENT, 50 ng of selectable marker and 250 ng of the non-selectable PCR construct were added. Cells were incubated statically with the transforming DNA (tDNA) for 5 hours at 30°C then grown out in 1 mL LB3 for 2 hours, serially diluted, and plated onto antibiotic plates. Transformations were additionally plated onto non-selective medium when calculating transformation efficiency.

To verify transformation products were incorporated into the genome, DNA was extracted from transformants, and additional rounds of PCR were performed. For selectable markers, PCR primers matching the homologous sequences facilitating SOE PCR were used to confirm the insertion of the antibiotic gene (Table S2). For the *oxyR* and 280 kb deletions, a process similar to multiplex allele-specific colony PCR was performed, as previously described (6). “Scar” primers were designed to amplify a ~600 bp or ~2 kb product if a deletion was successful and a ~1.6 kb or ~282 kb region if not, respectively. For *oxyR* complementation, primers amplified a ~1.8 kb product if the insertion was successful and a ~1.5 kb product if the insertion was unsuccessful (Table S2). Transformants were cryopreserved in a 96-well microtiter plate at −80°C in LB + 10% glycerol and thawed for subsequent screening. To rapidly screen isolates for incorporation of tDNA, PCR was first performed on pools of nine colonies. Colonies were added to 100 µL of sterile water and incubated at 90°C for 10 minutes. Cell debris was pelleted by centrifugation, and the supernatant was used as the DNA template in PCR. Individual colony PCR was performed subsequently to identify specific isolates that incorporated the tDNA. Colony PCR reactions were performed using Gotaq polymerase (Promega) according to the manufacturer’s protocols using thermocycler conditions as follows: (i) 95°C – 2 minutes, (ii) 95°C – 1 minute, (iii) 55°C – 1 minute, and (iv) 72°C – 1 minute/kb, repeat steps 2–4 25–30×, 72°C – 10 minutes, 4°C – hold. Products were visualized by Midori Green (Bulldog Bio) stain after gel electrophoresis (1.5% agarose). To confirm the deletion via Sanger sequencing, DNA was extracted using the DNeasy Blood and Tissue Kit (Qiagen) according to the manufacturer’s protocol for Gram-negative bacteria. PCR of the deletion scar was performed using Phusion Plus Polymerase according to the manufacturer’s protocol. Thermocycler conditions were as follows: (i) 98°C – 30 seconds, (ii) 98°C – 10 seconds, (iii) 60°C – 10 seconds, and (iv) 72°C – 30 seconds/kb, repeat steps 2–4 25–30×, 72°C – 5 minutes, 4°C – hold. The PCR reaction was purified with QIAquick PCR Purification Kit (Qiagen), and Sanger sequencing was performed by the UT Genomics Core or Eurofins Genomics.

### pMMB*sacBtfoX* plasmid curing

To cure *V. natriegens* Δ280 (EZ284) of pMMB*sacBtfoX*, cells were grown overnight in LB3, serially diluted, and plated onto LB0.1 (LB with 0.1% NaCl) supplemented with 15% sucrose. Plates were incubated at 30°C. Single colonies from sucrose-containing plates were transferred with sterile toothpicks to non-selective plates and kanamycin plates to confirm the loss of the plasmid via kanamycin sensitivity. Colonies that were both able to grow on sucrose and sensitive to kanamycin were considered putatively cured of the plasmid. PCR targeting the plasmid origin of replication was subsequently performed to confirm the loss of the entire plasmid rather than just the loss of the *sacB* and kan^R^ genes. DNA was extracted using the DNeasy Blood and Tissue Kit (Qiagen) following instructions for Gram-negative bacteria, and PCR was performed using Phusion Plus Polymerase according to the manufacturer’s protocols, using primers internal to the plasmid origin (Table S2). Thermocycler conditions were as follows: (i) 98°C – 30 seconds, (ii) 98°C – 10 seconds, (iii) 60°C – 10 seconds, (iv) 72°C – 30 seconds/kb, repeat steps 2–4 25–30×, 72°C – 5 minutes, 4°C – hold. The same process was used to create a plasmid-cured strain of *V. natriegens* ATCC 14048 harboring the spec^R^ marker (EZ278).

### Fitness assays

*V. natriegens* ATCC 14048 pMMB*tfoX* was used as the WT strain in fitness assays compared to strains marked with erm^R^ (EZ262) or spec^R^ (EZ263). Strains were grown overnight in 3 mL of LB3 at 30°C, shaking (250 rpm). Overnight cultures were diluted 1:1,000 into 3 mL of fresh LB3 medium. Growth curves of monocultures were obtained by viable count assay on a non-selective LB3 medium. Cocultures of WT and antibiotic-resistant (Ab^R^) strains were established by inoculating 3 µL of each overnight culture together into 3 mL LB3 and were titered onto both non-selective and antibiotic selection plates. CFU/mL of Ab^R^ cells was calculated from growth on relevant antibiotic plates. CFU/mL of WT cells was calculated by subtracting the CFU/mL of Ab^R^ cells from the total CFU/mL calculated on non-selective plates.

Complementation of *ΔoxyR* was assessed by comparing the survival of WT (EZ262), *ΔoxyR* (EZ274), and the *ΔoxyR* complement strain (EZ292) after exposure to 1.5 µM hydrogen peroxide in marine heterotroph minimal media [MHM; AMP-A with 10× trace metals (18–20)]. Strains were grown overnight in MHM + 1% acetate at 30°C, shaking (250 rpm), diluted 1:1,000, and grown overnight again. Cells were pelleted and washed three times in MHM and diluted 1:1,000 into 5 mL of fresh MHM. Cultures were incubated at 30°C, shaking (250 rpm) for 24 hours, then exposed to 1.5 µM hydrogen peroxide. Cell abundances were measured by viable count assay.

Plasmid-cured strains of WT *V. natriegens* ATCC 14048 marked with spec^R^ (EZ278) and the Δ280 kb mutant marked with erm^R^ (EZ289) were assayed for relative growth. Overnight cultures were diluted 1:1,000 in 10 mL LB3 and grown to an optical density (OD_600_) of ~0.2. Each culture was then diluted back to an OD_600_ of 0.1 and inoculated 1:100 in triplicate into test tubes with 3 mL LB3. Cultures were titered onto LB3 plates to calculate CFU/mL. Growth rates were calculated as the growth constant from the regression of cell number over time of four consecutive time points during exponential growth.

## RESULTS

### A prophage region can serve as a neutral site for antibiotic-resistance gene insertion

In the study that adapted MuGENT from *V. cholerae* to *V. natriegens*, the *dns* locus was chosen as the site for insertion of the kanamycin- or spectinomycin-resistance marker because loss of *dns* did not impact growth or viability in rich medium (6). Notably, loss of *dns*, which encodes an extracellular/periplasmic endonuclease, did not affect MuGENT transformation efficiency in *V. cholerae* (14, 21). While the Δ*dns*::kan^R^ and Δ*dns*::spec^R^ mutations showed no obvious fitness defect in rich medium in the *V. natriegens* study, we sought to identify a new location for selectable marker insertion with minimal impact on gene expression and physiology and may prove selectively neutral under most or all experimental conditions. Iterative mutagenesis with MuGENT requires two different drug-resistance markers (see below), which would normally be satisfied with the kan^R^ and spec^R^ markers used previously. However, because the *sacB* curable derivative of the transformation plasmid (see below) confers kanamycin resistance, we substituted this marker with a new one for MuGENT: erythromycin resistance (erm^R^).

**Fig 1:**
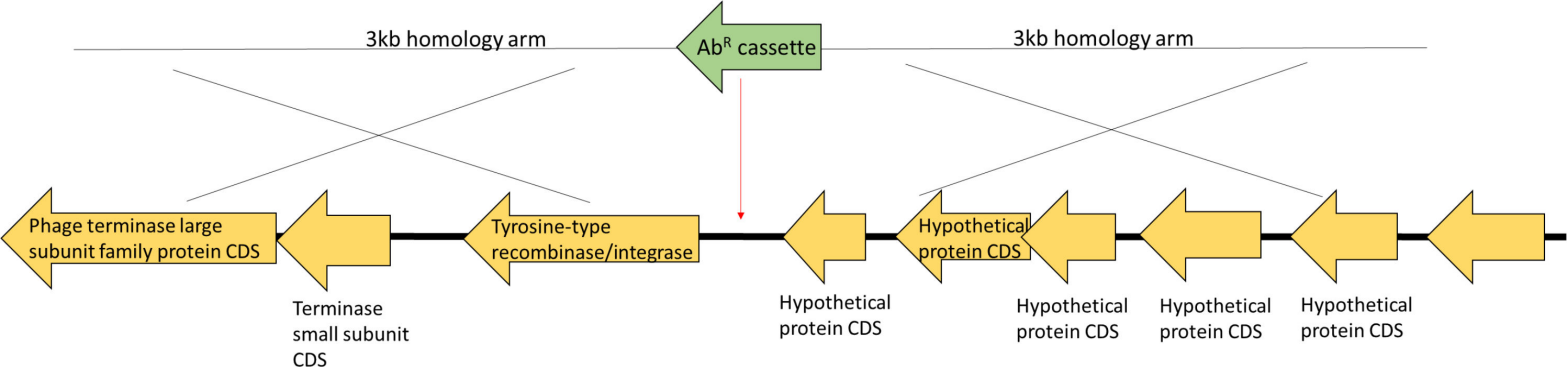
Location of a neutral site within a prophage region for antibiotic resistance marker insertion.

**Fig 2:**
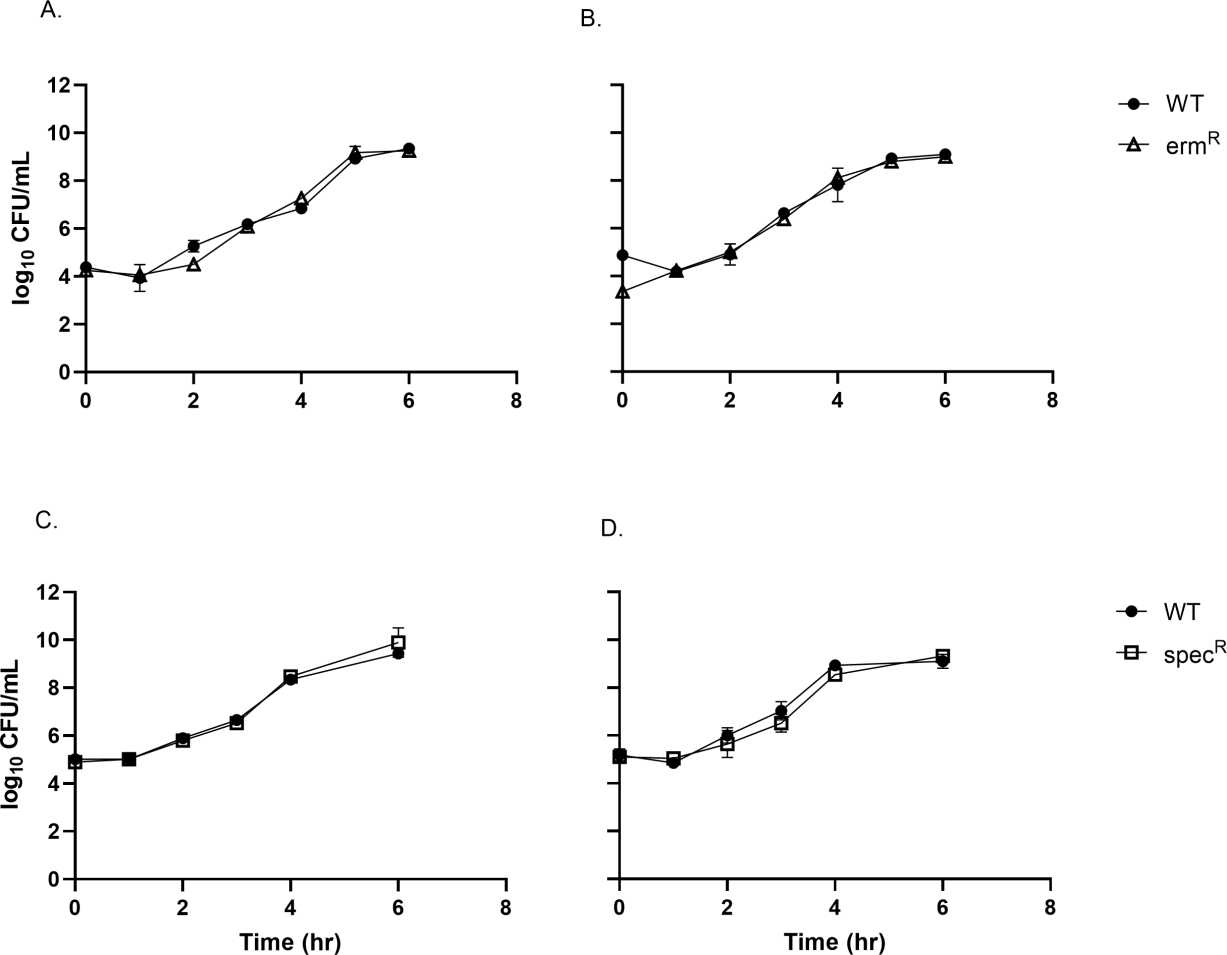
Antibiotic resistance gene insertion into a prophage region results in no growth defects. Growth of WT and antibiotic-resistant marked strains of *V. natriegens* in monocultures (**A and C**) and cocultures (**B and D**) in LB3 at 30°C (*n* = 3; ±SD of the geometric mean).

Two genomic locations on Chromosome 1 were tested as potential targets. Neutrality was defined as the absence of growth defects in the Ab^R^ marked strains compared to WT. The first insertion site was an intergenic region between two genes (HD-GYP domain-containing protein CDS; locus tag PN96_RS00670, and methyl-accepting chemotaxis protein CDS; locus tag PN96_RS00745), transcribed in opposing directions (Fig. S1A), reasoning that insertion into this location would avoid disruption of any intergenic gene regulation elements. However, unexpectedly, the insertion of an erm^R^ gene into this site resulted in a growth defect (Fig. S1B). This result highlights the unknown aspects of *V. natriegens* genome and gene regulation. The second insertion site was located within a prophage region between two genes (tyrosine-type recombinase/integrase CDS; locus tag PN96_RS07070 and hypothetical protein CDS; locus tag PN96_RS07075; Fig. 1), reasoning that this location would not disrupt fitness impacting processes of the cell. Insertion into the prophage region yielded no fitness defects from erm^R^ or spec^R^ gene insertions when the strains were grown in monoculture or cocultured with wild-type cells in a rich medium (Fig. 2A and B, respectively).

### Iterative MuGENT mutagenesis via marker swapping

Multiple rounds of engineering via MuGENT are possible so long as each selection event utilizes a different resistance marker than that of the prior selection. One means to facilitate endless iterations of MuGENT transformation is to simply alternate back and forth between two marker genes that replace each other via homologous recombination of flanking DNA at the same neutral site. To test this approach at the neutral site described above, transformation efficiencies were first calculated when either erm^R^ or spec^R^ markers were introduced into WT. Subsequently, transformation efficiencies were determined for replacing one marker for the other.

**Fig 3:**
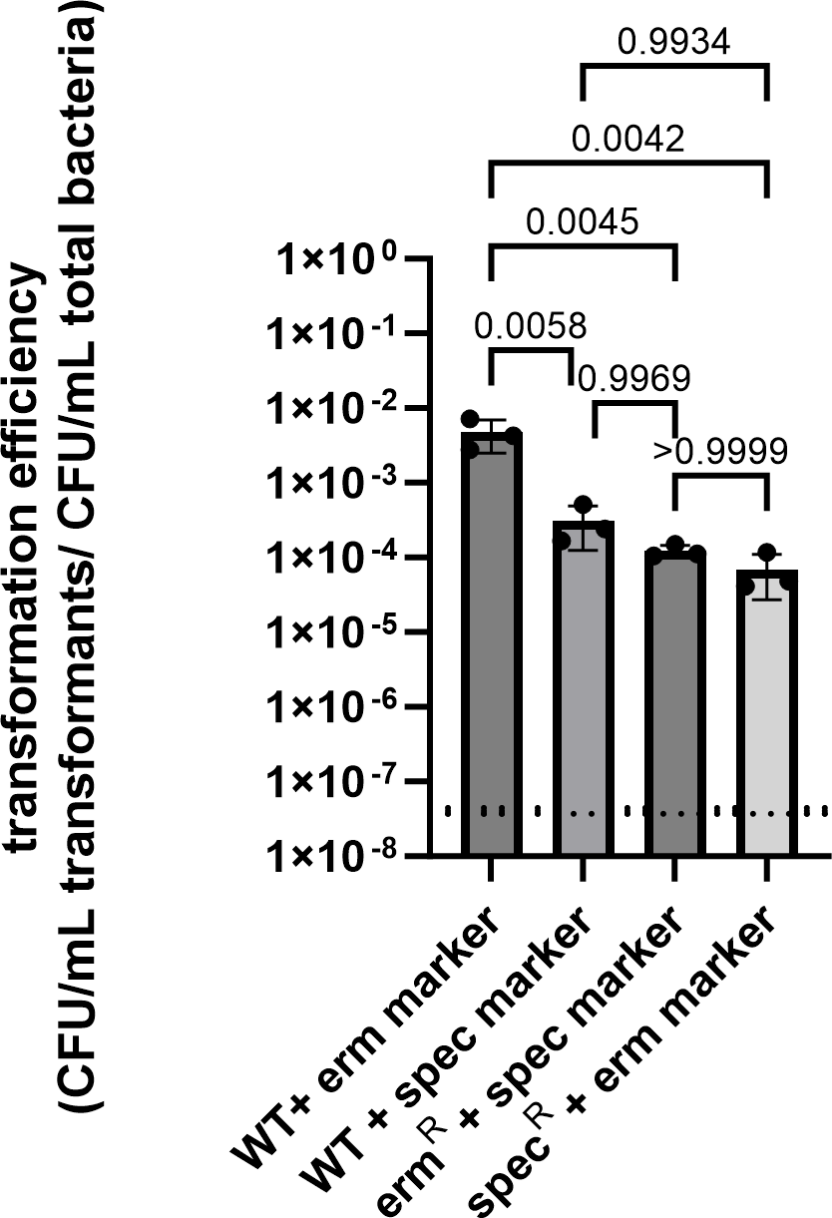
Antibiotic marker swapping efficiency. Efficiencies were calculated as CFU/mL on antibiotic-containing plates/CFU/mL on non-selective plates. *P*-values were calculated from three biological replicates using a one-way ANOVA multiple comparisons test with Tukey’s correction. The dashed line indicates the limit of detection calculated as the ratio of CFU/mL of negative control transformations (no tDNA added) on antibiotic-containing plates vs negative control transformations on non-selective plates. There were no colonies obtained from negative control transformations plated on 100 µg/mL erythromycin plates; however, it was found that there is a low rate (4.5–9.7 × 10^−8^) of spontaneous spectinomycin resistance that arises when the negative control mock transformations are plated on media containing 250 µg/mL spectinomycin.

Rates of marker replacement were sufficiently high suggesting that this approach can facilitate multiple rounds of mutation via MuGENT (Fig. 3). Notably, however, the transformation efficiency of insertion of the erm^R^ marker into WT was significantly higher than all others. Interestingly, the first round of transformation with either selectable marker required extended incubation, 24–36 hours, to form full-sized colonies. Whereas, subsequent swapping of the markers required ≤16–18 hours for colonies to fully form. All strains grew normally after initial isolation (Fig. 2A and B).

### Chromosomal complementation of *oxyR*

**Fig 4:**
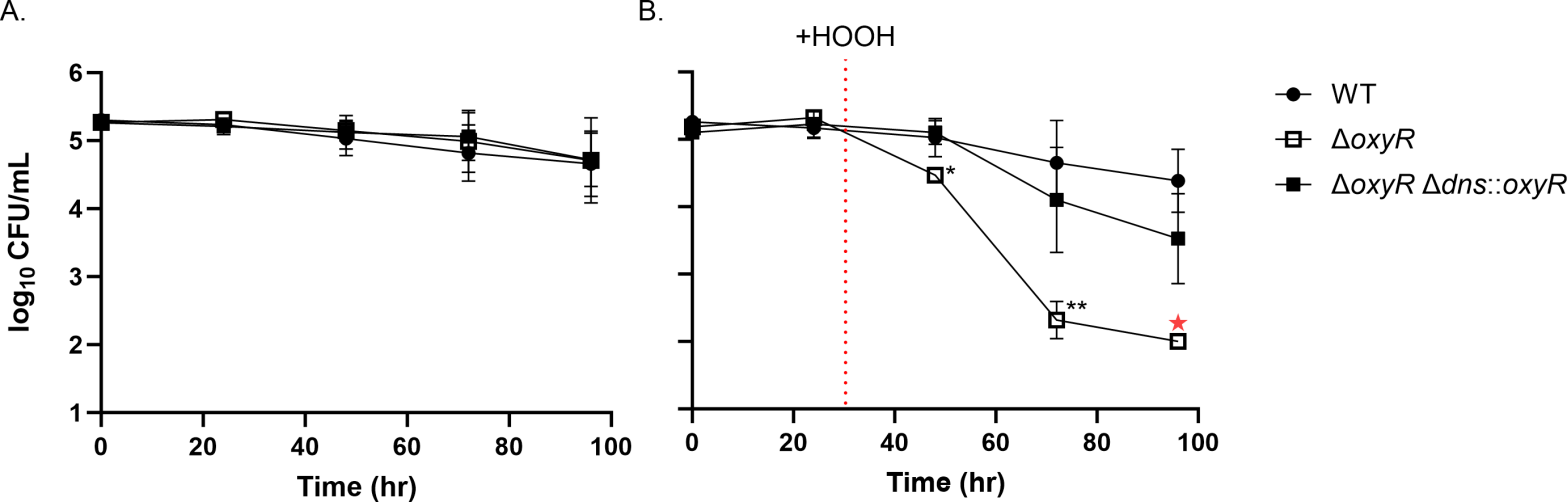
Chromosomal complementation of Δ*oxyR* rescues cells exposed to hydrogen peroxide. Cell viability of WT, Δ*oxyR*, and Δ*oxyR* Δ*dns::oxyR* in MHM without hydrogen peroxide (HOOH) (**A**), and with exposure to HOOH (**B**). Red dashed line indicates the addition of 1.5 µM HOOH. Red star indicates cell densities below the limit of detection (<100 CFU/mL). *P*-values were calculated from three biological replicates using a one-way ANOVA multiple comparisons test with Dunnett’s correction, *P ≤ 0.05 and **P ≤ 0.01.

Complementation of deletion mutations in *V. natriegens* has been achieved by plasmid-based expression systems and reversion mutations (14, 22, 23). However, to our knowledge, the complementation of a deleted gene by insertion of that gene at an ectopic chromosomal locus has not yet been developed. To assess this, we created a knockout mutant of *oxyR*, Δ*oxyR* (EZ274)*,* which resulted in cells with an increased sensitivity to hydrogen peroxide compared to WT (EZ262; Fig. 4B), consistent with studies in other bacteria (24–26). We then complemented Δ*oxyR* by replacing the *dns* open-reading frame and promoter region (−138 to −1 bp) with those of *oxyR* (promoter region = −124 to −1 bp), creating Δ*oxyR* Δ*dns::oxyR* (EZ292). This construct includes 50 bp of the 3’ UTR of *oxyR* placed upstream of the 3’ UTR of *dns*. We reasoned that this site has previously been used for selectable marker insertion (see above) and could be used to validate the concept of chromosomal complementation. In the absence of hydrogen peroxide, cell viability of Δ*oxyR* and Δ*oxyR* Δdns::*oxyR* did not differ from WT (Fig. 4A). Whereas, Δ*oxyR* abundance declined rapidly and fell below the limit of detection (100 cells/mL) within 72 hours of hydrogen peroxide exposure, while Δ*oxyR* Δ*dns::oxyR* retained cell counts similar to WT (Fig. 4B). Notably, a small but not statistically significant decrease in Δ*oxyR* Δ*dns::oxyR* counts relative to WT was observed by 72 hours after hydrogen peroxide exposure.

### Natural transformation can be used to create deletions up to 280 kb in the genome of *V. natriegens* in a single round of mutagenesis

**Fig 5:**
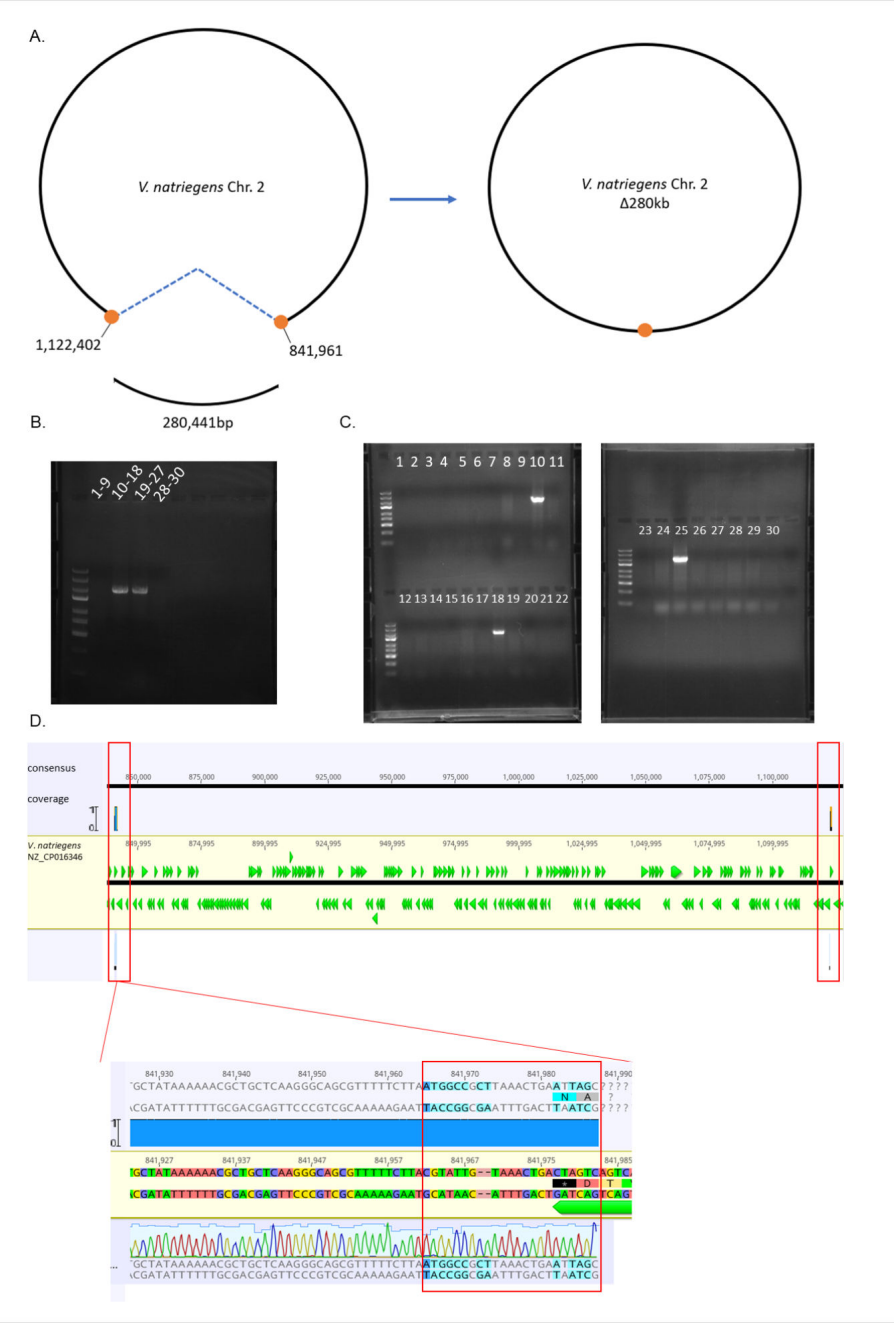
Homologous recombination achieves deletions up to 280 kb. **(A**) Schematic depicts the location at which a 280,441 bp deletion was created. Orange circles represent the 3 kb amplicons that were adjoined through PCR to create the construct used in natural transformation. The blue dashed line represents the removal of the segment of DNA between the two deletion endpoints. In the resulting deletion strain, the two 3 kb amplicons are linked together by a 20–23 bp scar. (**B**) Grouped colony PCR identifies subsets of colonies containing the deletion. Number ranges indicate the isolate #’s groups together in the PCR. (**C**) Individual colony PCR confirms individual colonies containing the deletion. Numbers indicate the isolate # ran in each column. The Thermofisher Gene Ruler Express Ladder is used in all gels pictured. (**D**) Sanger sequencing of PCR product aligned to *V.natriegens* genome. Red boxes highlight that the sequence is mapped to the endpoints, indicating the two distal segments are now connected, and everything in between has been deleted. The zoomed-in image shows the presence of the 23 bp scar sequence (red box). Green arrows represent open reading frames, and right- and left-pointing arrows are encoded on the top and bottom stand, respectively.

MuGENT has been shown to be an incredibly efficient method for creating markerless point mutations, small indels, and single gene deletions (6, 12, 14, 27), but the maximum size of deletions has not been constrained. Chromosome 2 contains only six genes essential for growth in rich medium, and they are separated by large regions of non-essential genes (10), making the chromosome an ideal target for deletion analysis. For the initial attempt, we targeted a 280,441 bp region lacking essential genes (Fig. 5A). Natural transformation was used to co-transform the erm^R^ marker and Δ280-creating construct into *V. natriegens* pMMB*sacBtfoX*. Colonies were isolated on an erythromycin-containing medium following transformation and cryopreserved in a 96-well plate. To rapidly screen colonies for the deletion, PCR was first performed on pools of nine colonies using the deletion scar primers, 840291F and 1122735R (Table S2). This screen indicated two pools contained putative deletions of 280 kb (Fig. 5B). Colony PCR was performed for every isolate to verify the pooled colony result and to identify those individual isolates with the deletion (Fig. 5C). Individual screening results were consistent with the pooled screening result and identified that 3/30 of the colonies screened obtained the 280 kb deletion. Sanger sequencing confirmed the loss of the 280 kb and the presence of the scar (Fig. 5D). The deletion of 280 kb removed 239 genes including ones involved in transcriptional regulation, catabolism, iron transport, chemotaxis, oxidative stress, and osmotic stress (File S2).

To determine whether the size of the targeted deletion impacts mutation efficiency, we compared deletion rates for the 280 kb region (~10%, Fig. 5C) vs a single gene (2172 bp) that constituted one end of the 280 kb deletion region. The gene target was one of three copies of *katG* (locus tag BA890_RS19750). The natural transformation was performed exactly the same as with the 280 kb deletion, and 30 colonies (transformants that acquired at minimum the antibiotic resistance gene) were screened for *katG* deletion using flanking primers (Table S2). Of the 30 colonies screened, nine showed the deletion (Fig. S2). Therefore, co-transformation to create a 280 kb deletion was roughly three times less efficient than a 2 kb deletion sharing one deletion end, though the lower rate was still well within the scope of feasibility by the MuGENT technique.

### *sacB* counter-selection permits curing of competence plasmid pMMB*sacBtfoX* from *V. natriegens*

Natural transformation in *V. natriegens* is dependent on plasmid-borne expression of a competence regulator gene, *tfoX*. Being able to cure the plasmid is beneficial for downstream analysis when it is no longer needed, as maintaining a multi-copy plasmid may lower fitness. However, *V. natriegens* is quite recalcitrant to losing the plasmid, and a robust curing technique has not yet been developed for this organism. One report indicated successful curing by strongly inducing *tfox* expression to increase metabolic burden and promote plasmid loss; however, screening efficiency for this method was not reported (28). It has recently been shown that the addition of a *sacB* gene onto pMMB*tfox* could be used to cure the plasmid from *V. parahaemolyticus* via sucrose counterselection (12). We tested whether this *sacB* harboring plasmid, pMMB*sacBtfoX*, can also be lost from *V. natriegens* by sucrose counterselection.

**Fig 6:**
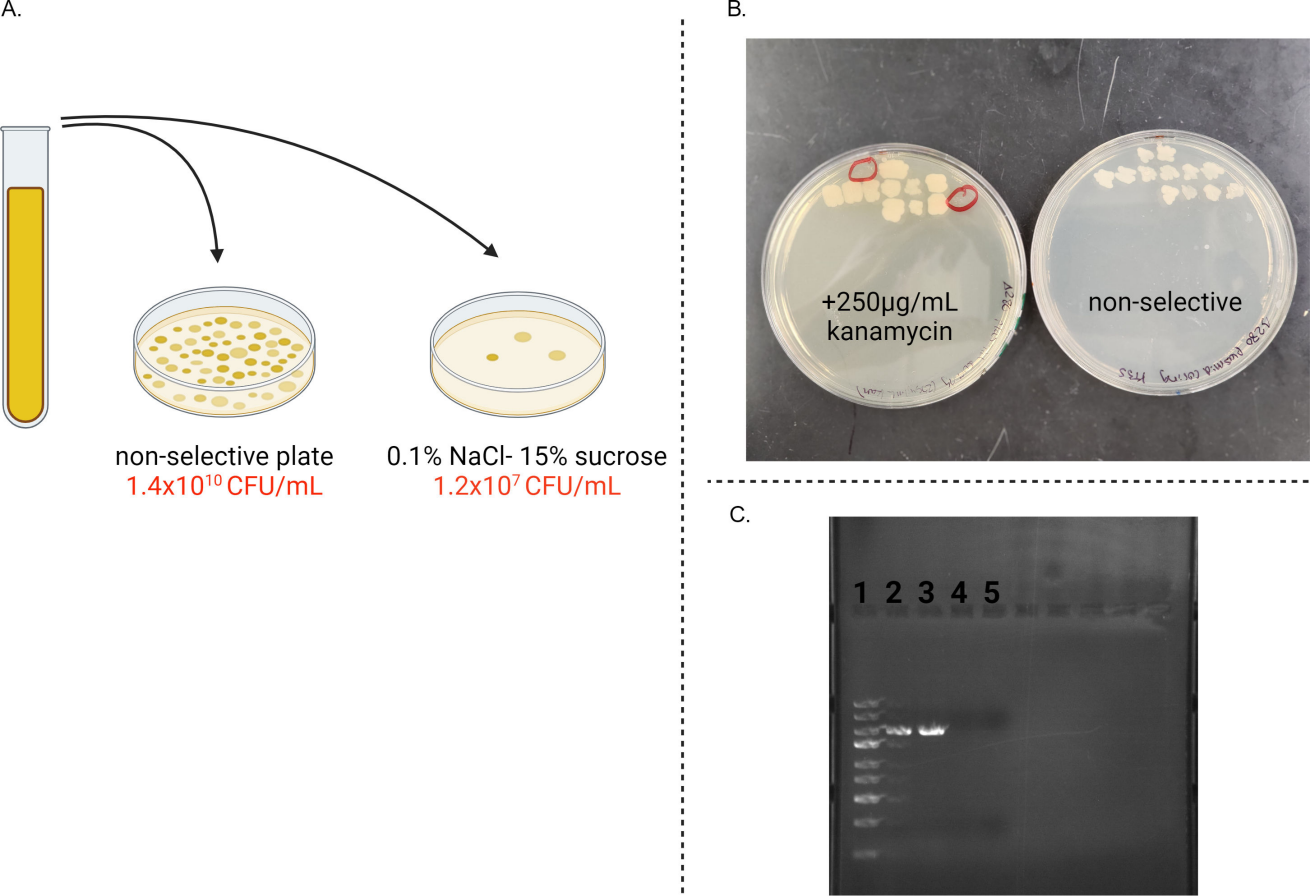
Plasmid curing accomplished with *sacB-*mediated selection. (**A**) CFU/mL calculation of Δ280-pMMB*sacBtfox* bacterial culture plated on non-selective and sucrose-containing media. (**B**) Growth of individual colonies from sucrose-containing plate on non-selective and kanamycin-containing plates. Red circles highlight the colonies that were kanamycin sensitive. (**C**) DNA was extracted from both sucrose^R^- kanamycin^s^ strains. Thermofisher GeneRuler express ladder (column 1), PCR amplification of the Δ280 scar (columns 2 and 3) and the plasmid *ori* (columns 4 and 5) were performed to confirm loss of the plasmid. Created with Biorender.com.

After the pMMB*sacBtfoX* plasmid was used to create the Δ280 kb mutant, we implemented counter-selection on sucrose for plasmid curing. Successful curing was achieved by plating on a medium containing 15% sucrose and low salt, 0.1% NaCl (LB0.1 + 15% sucrose). The addition of 0.1% NaCl, while low, was found to be necessary for the growth of *V. natriegens* on these plates. Plating cultures on LB0.1 + 15% sucrose resulted in a 1,000-fold reduction in the number of colonies compared to growth on non-selective medium (Fig. 6A). Single colonies on LB0.1 + 15% sucrose were transferred to LB3 (no sucrose) plates containing kanamycin to confirm loss of the kan^R^-conferring plasmid. Some colonies showed the desired kan^S^ phenotype; however, some colonies retained kanamycin resistance (Fig. 6B). This indicated that the sucrose selection allows for some false positives to arise. For the putative cured sucrose^R^-kanamycin^s^ colonies, DNA extraction and PCR amplification of the plasmid *ori* further confirmed loss of the plasmid (Fig. 6C). It should be emphasized that the reduced salt content of the medium was essential for sucrose counterselection: in full strength (3%) salt media no reduction in colony formation was seen, even in the presence of 15% sucrose, and no kanamycin^s^ colonies were found.

### Deletion of 280 kb impacts cell growth in rich media

**Fig 7:**
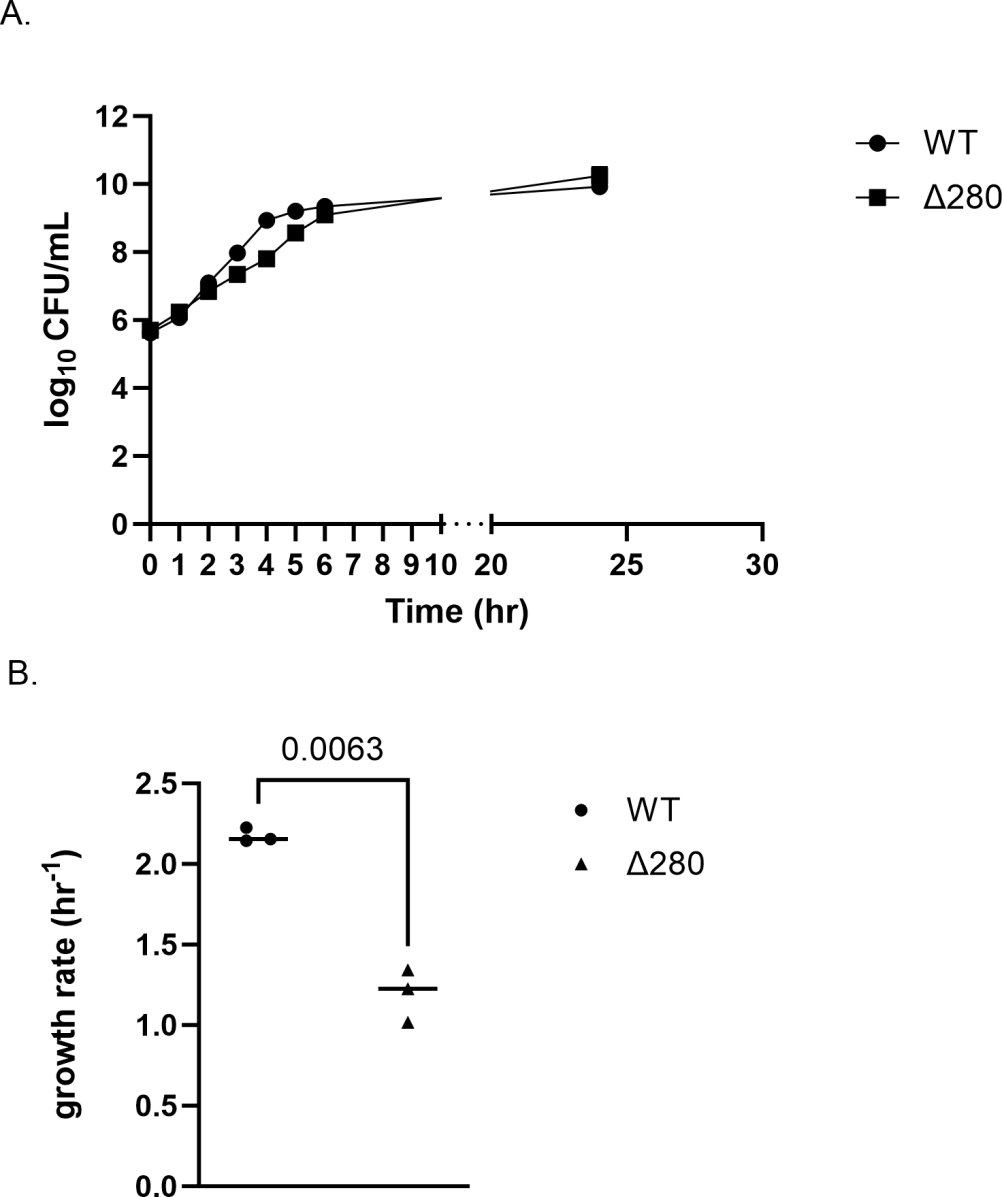
Fitness of Δ280 kb strain. (A) Monoculture growth curves and (B) growth rates (hour^−1^) of WT and Δ280 kb strains of *V. natriegens* in LB3 medium at 30°C (*n* = 3; ±SD of the geometric mean). *P*-value was calculated using a two-tailed, unpaired *t* test with Welch’s correction.

To assess possible growth defects of the Δ280 kb deletion, we compared monoculture growth curves for plasmid-cured strains of Δ280 kb (EZ289) marked with erm^R^ and WT *V. natriegens* marked with spec^R^ (EZ278). In LB3 medium, Δ280 (EZ289) achieved a lower maximum growth rate than WT but both strains reached similar final abundances (Fig. 7B and A, respectively). Thus, despite the removal of 239 genes*,* the deletion mutant remained viable and had the same growth yield as WT with only a small defect in growth rate.

## DISCUSSION

This work expands the genetic toolset for *V. natriegens* and advances the genomic engineering capabilities of this emerging model organism. We identified a neutral site for selectable marker insertion within a prophage region of Chromosome 1, providing an alternative target that does not require disruption of the *dns* gene. Additionally, we demonstrated the ability to select for transformants in iterative rounds of MuGENT with high efficiency via marker swapping at each round. Here, we implemented selectable markers establishing erythromycin or spectinomycin resistance, but a number of antibiotics have been used for the selection of *V. natriegens* after transformation (29) and should be feasible to use. We further applied this to create a knockout mutant of *oxyR*, and subsequent insertion of *oxyR* at a different locus, and we showed that chromosomal complementation permits phenotypic rescue of *oxyR* mutants. To demonstrate the ability of MuGENT to make massive edits to the genomic chassis in single transformations, we created a 280 kb markerless deletion, removing 239 genes from the genome with some loss of maximum growth rate but no apparent decrease in cell yield. Finally, as a useful cleanup step after mutagenesis, we established a protocol for curing the transformants of the plasmid necessary for MuGENT using the *sacB* counterselection method.

The new marker insertion site is found within a prophage region on Chromosome 1. It should be noted that a previous study has characterized this prophage region, finding that excision can be induced by DNA damaging agent, mitomycin C, and it can undergo spontaneous induction, but at a low rate of <1% in exponentially growing cells and 0.001% in stationary phase cells (11). Prophage induction could theoretically lead to marker loss from the chromosome, but induction should culminate in cell lysis and mortality, and in either case, loss of drug resistance can be screened readily.

Notably, our transformation efficiencies were two- to threefold lower than previously reported (6), and the initial insertion of either marker at the prophage locus resulted in a longer incubation time (24–36 hours) for colonies to fully form. The cause of this delay is unknown, but perhaps the insertion of the selectable marker triggers an elevated rate of spontaneous induction that slows colony enlargement through the release and lytic infection by the phage within the growing population. Alternatively, there could be a lag in the expression of the selectable marker, which would result in some initial cell death and thus slower colony formation. However, and importantly, subsequent culturing of the strains harboring selectable markers showed no difference in fitness compared to wild-type cells and in iterative rounds of transformation that replace one marker with another, colonies form normally (16–18 hours). Finally, the initial insertion of the erythromycin marker into WT had a significantly higher transformation efficiency than all other marker insertions. It is notable that the erythromycin marker used here has also been described as a superior marker for use in *Vibrio fischeri* because it resulted in higher recovery of transconjugants and lower rates of spontaneous resistance (30). While several antibiotics have been shown to work as selective markers in *V. natriegens*, future studies could benefit from a comprehensive understanding of marker efficiencies to optimize mutagenesis protocols in *V. natriegens* and other *Vibrio* species.

Deletion of *oxyR* resulted in hypersensitivity to hydrogen peroxide, indicating a likely familiar role in activation of antioxidant genes during oxidative stress (31). It should be noted that this phenotype was observed after 24-hour acclimation in a minimal medium and exposure to 1.5 µM hydrogen peroxide. These conditions were chosen to more closely mimic environmental conditions than the rich medium typically used for laboratory culturing and proved useful for exhibiting a detectable phenotype.

Chromosomal complementation of *ΔoxyR* was successful in rescuing the deletion phenotype, demonstrating the feasibility of single-copy, chromosomal complementation. We used the *dns* gene as the location for this ectopic expression, providing the native promoter region of *oxyR* and leaving the 3’ UTR of *dns* intact. This method provides an alternative to plasmid-based complementation and can be useful when plasmid maintenance and/or expression of genes on multicopy plasmids are undesired.

Higher growth rate (32), cell density (32, 33), recombinant protein production (32–35), improved genetic stability (32, 36), and greater DNA uptake efficiency (36) have all been observed in deletion constructs of bacteria with bioproduction potential [see also reviews (37, 38)]. As such, much effort has been given to investigating the effects of genome reduction, the minimal set of genes required for life, and to creating a reduced chassis desirable for bioproduction applications, as recently reviewed (39, 40). One major challenge in these areas is the labor-intensive process to create deletions. Our work illustrates the ease of using *V. natriegens* and provides a framework to apply *V. natriegens* in the aforementioned areas of research. Here, we have demonstrated the ability to delete 280 kb of DNA in a single round of targeted mutagenesis. To our knowledge, this is the largest deletion made by MuGENT and one of the largest deletions made in a single round of mutagenesis of a bacterial chromosome (41). The upper bound of possible deletion size using this methodology is unknown but presumably set by the maximum distance between essential genes. Chromosome 2 of *V. natriegens* contains regions >500 kb lacking any predicted essential genes, so future studies may successfully generate deletions larger than 280 kb. However, challenges facing the creation of larger deletions using this method may include the physical distance between recombination sites and the DNA topology of the chromosome that the tDNA must interact with for recombination to occur. Additionally, the definition of “essentiality” should be considered. Caution should be taken as there are additional “growth supporting” genes (required for rapid growth in rich medium) throughout the chromosome (10). The deletion of growth-supporting genes in our 280 kb deletion (e.g., *pyrC*, a DEAD/DEAH box helicase, a hypothetical protein, an Sco family protein, and an iron chelate uptake ABC transporter family permease subunit) likely contributed to the growth defect we observed. Consideration should be given to growth conditions that may impact gene essentiality and to the possibility of conditionally lethal genes, that may impede multi-gene deletions (42). Furthermore, it has been shown that deletions of “dispensable” genomic loci sometimes result in unexpected negative effects (35, 43, 44).

Our pooled colony screening technique proved useful to rapidly screen multiple transformation isolates simultaneously, with 100% accuracy of identifying pools that contained isolates with our targeted deletions. We also found that deletion of 280 kb occurred in 10% of colonies screened, while deletion of a single gene (2 kb) occurred in 30% of colonies screened. Both the size of PCR constructs to create these deletions and the transformation protocols used were performed the same for both deletion lengths. One simple interpretation of this difference is that the proximities of the two deletion ends are different for the two construct sizes due to chromosomal packaging, and this proximity contributes to overall recombination efficiency. Another non-mutually exclusive explanation is that the gene function(s) lost in the large deletion contribute in some way to (initial) colony formation, though subsequent rounds of growth show no loss in plating efficiency for the deletion mutant.

Finally, we report the successful implementation of *sacB*-mediated plasmid curing in *V. natriegens*. This cleanup step removes the plasmid from the host genome, decreasing the metabolic burden on the host and removing the necessity of maintaining the culture under antibiotic selection to ensure genetic homogeneity between treatment groups in downstream analysis of the mutants (i.e., all groups should have the plasmid, or not have the plasmid, to prevent ambiguity that can occur if spontaneous curing happens in absence of selection). Plasmid curing was only achievable when we reduced salt from the selection media. We hypothesize that the reduction in salt concentration improved *sacB*-mediated cell death by enriching the osmolyte pool with sucrose: addition by subtraction. Similarly, reducing or removing salt entirely from the media has been shown to increase sucrose sensitivity in *sacB*-harboring cells of *E. coli* K12 and *Burkholderia pseudomallei* (45, 46), but to our knowledge, the mechanisms behind this have not been experimentally determined. In *B. subtilis,* it has also been shown that NaCl influences the expression of *sacB*; however, it was found that higher NaCl concentrations induced higher expression of *sacB* (47), suggesting a different mechanism may be responsible for the improved counter-selection phenotype seen here. Importantly, this method worked only when salt was reduced but not eliminated. Elimination of salt prevented the growth of *V. natriegens* in our media, which is consistent with prior reports that substrate uptake and cell proliferation by this organism are dependent on the availability of salt ions (2, 48, 49).

## Data Availability

The data underlying this article are available in the article and in its online supplementary material.

## SUPPLEMENTAL MATERIAL

Supplemental figures and tables spectrum.03964-23-s0001.pdf Fig. S1 and S2; Tables S1 and S2.

Supplemental material spectrum.03964-23-s0002.xlsx File S2.

ASM does not own the copyrights to Supplemental Material that may be linked to, or accessed through, an article. The authors have granted ASM a non-exclusive, world-wide license to publish the Supplemental Material files. Please contact the corresponding author directly for reuse.

## References

[1] Payne WJ, Eagon RG, Williams AK. 1961. Some observations on the physiology of Pseudomonas natriegens nov. spec. Antonie Van Leeuwenhoek 27:121–128. doi:10.1007/BF02538432 13733692

[2] Payne WJ. 1958. Studies on bacterial utilization of uronic acids III. induction of oxidative enzymes in a marine isolate. J Bacteriol 76:301–307. doi:10.1128/jb.76.3.301-307.1958 13575389 PMC290205

[3] Eagon RG. 1962. Pseudomonas natriegens, a marine bacterium with a generation time of less than 10 minutes. J Bacteriol 83:736–737. doi:10.1128/jb.83.4.736-737.1962 13888946 PMC279347

[4] Lee HH, et al.. 2016. Vibrio natriegens, a new genomic powerhouse. bioRxiv. doi:10.1101/058487

[5] Weinstock MT, Hesek ED, Wilson CM, Gibson DG. 2016. Vibrio natriegens as a fast-growing host for molecular biology. Nat Methods 13:849–851. doi:10.1038/nmeth.3970 27571549

[6] Dalia TN, Hayes CA, Stolyar S, Marx CJ, McKinlay JB, Dalia AB. 2017. Multiplex genome editing by natural transformation (MuGENT) for synthetic biology in Vibrio natriegens. ACS Synth Biol 6:1650–1655. doi:10.1021/acssynbio.7b00116 28571309 PMC6519440

[7] Zhang Y, Li Z, Liu Y, Cen X, Liu D, Chen Z. 2021. Systems metabolic engineering of Vibrio natriegens for the production of 1, 3-propanediol. Metab Eng 65:52–65. doi:10.1016/j.ymben.2021.03.008 33722653

[8] Wang Z, Tschirhart T, Schultzhaus Z, Kelly EE, Chen A, Oh E, Nag O, Glaser ER, Kim E, Lloyd PF, Charles PT, Li W, Leary D, Compton J, Phillips DA, Dhinojwala A, Payne GF, Vora GJ. 2020. Melanin produced by the fast-growing marine bacterium Vibrio natriegens through heterologous biosynthesis: characterization and application. Appl Environ Microbiol 86:e02749-19. doi:10.1128/AEM.02749-19 31836580 PMC7028964

[9] Hoffart E, Grenz S, Lange J, Nitschel R, Müller F, Schwentner A, Feith A, Lenfers-Lücker M, Takors R, Blombach B. 2017. High substrate uptake rates empower Vibrio natriegens as production host for industrial biotechnology. Appl Environ Microbiol 83:e01614-17. doi:10.1128/AEM.01614-17 28887417 PMC5666143

[10] Lee HH, Ostrov N, Wong BG, Gold MA, Khalil AS, Church GM. 2019. Functional genomics of the rapidly replicating bacterium Vibrio natriegens by CRISPRi. Nat Microbiol 4:1105–1113. doi:10.1038/s41564-019-0423-8 30962569

[11] Pfeifer E, Michniewski S, Gätgens C, Münch E, Müller F, Polen T, Millard A, Blombach B, Frunzke J. 2019. Generation of a prophage-free variant of the fast-growing bacterium Vibrio natriegens. Appl Environ Microbiol 85:e00853-19. doi:10.1128/AEM.00853-19 31253674 PMC6696956

[12] Chimalapati S, de Souza Santos M, Servage K, De Nisco NJ, Dalia AB, Orth K. 2018. Natural transformation in Vibrio parahaemolyticus: a rapid method to create genetic deletions. J Bacteriol 200:e00032-18. doi:10.1128/JB.00032-18 29555695 PMC6040184

[13] Lloyd CJ, Mejia-Santana A, Dalia TN, Dalia AB, Klose KE. 2021. Natural transformation in a classical-biotype Vibrio cholerae strain. Appl Environ Microbiol 87:e00060-21. doi:10.1128/AEM.00060-21 33712424 PMC8117766

[14] Dalia TN, Yoon SH, Galli E, Barre F-X, Waters CM, Dalia AB. 2017. Enhancing multiplex genome editing by natural transformation (MuGENT) via inactivation of ssDNA exonucleases. Nucleic Acids Res 45:7527–7537. doi:10.1093/nar/gkx496 28575400 PMC5499599

[15] Zheng M, Storz G. 2000. Redox sensing by prokaryotic transcription factors. Biochem Pharmacol 59:1–6. doi:10.1016/s0006-2952(99)00289-0 10605928

[16] De Souza Silva O, Blokesch M. 2010. Genetic manipulation of Vibrio cholerae by combining natural transformation with FLP recombination. Plasmid 64:186–195. doi:10.1016/j.plasmid.2010.08.001 20709100

[17] Saltikov CW, Newman DK. 2003. Genetic identification of a respiratory arsenate reductase. Proc Natl Acad Sci USA 100:10983–10988. doi:10.1073/pnas.1834303100 12939408 PMC196913

[18] Calfee BC, Glasgo LD, Zinser ER. 2022. Prochlorococcus exudate stimulates heterotrophic bacterial competition with rival phytoplankton for available nitrogen. mBio 13:e0257121. doi:10.1128/mbio.02571-21 35012332 PMC8749424

[19] Jeffrey Morris J, Zinser ER. 2013. Continuous hydrogen peroxide production by organic buffers in phytoplankton culture media. J Phycol 49:1223–1228. doi:10.1111/jpy.12123 27007639

[20] Moore LR, Coe A, Zinser ER, Saito MA, Sullivan MB, Lindell D, Frois‐Moniz K, Waterbury J, Chisholm SW. 2007. Culturing the marine cyanobacterium Prochlorococcus. Limnol Oceanogr : Methods 5:353–362. doi:10.4319/lom.2007.5.353

[21] Blokesch M, Schoolnik GK. 2008. The extracellular nuclease DNS and its role in natural transformation of Vibrio cholerae. J Bacteriol 190:7232–7240. doi:10.1128/JB.00959-08 18757542 PMC2580679

[22] Coppens L, Tschirhart T, Leary DH, Colston SM, Compton JR, Hervey WJ, Dana KL, Vora GJ, Bordel S, Ledesma-Amaro R. 2023. Vibrio natriegens genome‐scale modeling reveals insights into halophilic adaptations and resource allocation. Mol Syst Biol 19:e10523. doi:10.15252/msb.202110523 36847213 PMC10090949

[23] Zhang Y, Sun Q, Liu Y, Cen X, Liu D, Chen Z. 2021. Development of a plasmid stabilization system in Vibrio natriegens for the high production of 1,3-propanediol and 3-hydroxypropionate. Bioresour Bioprocess 8:1–11. doi:10.1186/s40643-021-00485-0 38650249 PMC10992974

[24] Christman MF, Morgan RW, Jacobson FS, Ames BN. 1985. Positive control of a regulon for defenses against oxidative stress and some heat-shock proteins in Salmonella typhimurium. Cell 41:753–762. doi:10.1016/s0092-8674(85)80056-8 2988786

[25] Greenberg JT, Demple B. 1988. Overproduction of peroxide‐scavenging enzymes in Escherichia coli suppresses spontaneous mutagenesis and sensitivity to redox‐cycling agents in oxyR‐mutants. EMBO J 7:2611–2617. doi:10.1002/j.1460-2075.1988.tb03111.x 2847922 PMC457135

[26] Kong I-S, Bates TC, Hülsmann A, Hassan H, Smith BE, Oliver JD. 2004. Role of catalase and oxyR in the viable but nonculturable state of Vibrio vulnificus. FEMS Microbiol Ecol 50:133–142. doi:10.1016/j.femsec.2004.06.004 19712354

[27] Dalia AB, McDonough E, Camilli A. 2014. Multiplex genome editing by natural transformation. Proc Natl Acad Sci USA 111:8937–8942. doi:10.1073/pnas.1406478111 24889608 PMC4066482

[28] Stukenberg D, Hensel T, Hoff J, Daniel B, Inckemann R, Tedeschi JN, Nousch F, Fritz G. 2021. The Marburg Collection: a Golden Gate DNA assembly framework for synthetic biology applications in Vibrio natriegens. ACS Synth Biol 10:1904–1919. doi:10.1021/acssynbio.1c00126 34255476

[29] Hoff J, Daniel B, Stukenberg D, Thuronyi BW, Waldminghaus T, Fritz G. 2020. Vibrio natriegens: an ultrafast-growing marine bacterium as emerging synthetic biology chassis. Environ Microbiol 22:4394–4408. doi:10.1111/1462-2920.15128 32537803

[30] Lyell NL, Dunn AK, Bose JL, Vescovi SL, Stabb EV. 2008. Effective mutagenesis of Vibrio fischeri by using hyperactive mini-Tn 5 derivatives. Appl Environ Microbiol 74:7059–7063. doi:10.1128/AEM.01330-08 18805998 PMC2583470

[31] Storz G, Tartaglia LA, Ames BN. 1990. The OxyR regulon. Antonie Van Leeuwenhoek 58:157–161. doi:10.1007/BF00548927 2256675

[32] Lieder S, Nikel PI, de Lorenzo V, Takors R. 2015. Genome reduction BOOSTS heterologous gene expression in Pseudomonas putida. Microb Cell Fact 14:23. doi:10.1186/s12934-015-0207-7 25890048 PMC4352270

[33] Mizoguchi H, Sawano Y, Kato J, Mori H. 2008. Superpositioning of deletions promotes growth of Escherichia coli with a reduced genome. DNA Res 15:277–284. doi:10.1093/dnares/dsn019 18753290 PMC2575892

[34] Park MK, Lee SH, Yang KS, Jung S-C, Lee JH, Kim SC. 2014. Enhancing recombinant protein production with an Escherichia coli host strain lacking insertion sequences. Appl Microbiol Biotechnol 98:6701–6713. doi:10.1007/s00253-014-5739-y 24752842

[35] Morimoto T, Kadoya R, Endo K, Tohata M, Sawada K, Liu S, Ozawa T, Kodama T, Kakeshita H, Kageyama Y, Manabe K, Kanaya S, Ara K, Ozaki K, Ogasawara N. 2008. Enhanced recombinant protein productivity by genome reduction in Bacillus subtilis. DNA Res 15:73–81. doi:10.1093/dnares/dsn002 18334513 PMC2650625

[36] Pósfai G, Plunkett G, Fehér T, Frisch D, Keil GM, Umenhoffer K, Kolisnychenko V, Stahl B, Sharma SS, de Arruda M, Burland V, Harcum SW, Blattner FR. 2006. Emergent properties of reduced-genome Escherichia coli. Science 312:1044–1046. doi:10.1126/science.1126439 16645050

[37] Choe D, Cho S, Kim SC, Cho B-K. 2016. Minimal genome: worthwhile or worthless efforts toward being smaller. Biotechnol J 11:199–211. doi:10.1002/biot.201400838 26356135

[38] Ma S, Su T, Lu X, Qi Q. 2023. Bacterial genome reduction for optimal chassis of synthetic biology: a review. Crit Rev Biotechnol:1–14. doi:10.1080/07388551.2023.2208285 37380345

[39] LeBlanc N, Charles TC. 2022. Bacterial genome reductions: tools, applications, and challenges. Front Genome Ed 4:957289. doi:10.3389/fgeed.2022.957289 36120530 PMC9473318

[40] Kurokawa M, Ying BW. 2019. Experimental challenges for reduced genomes: the cell model Escherichia coli. Microorganisms 8:3. doi:10.3390/microorganisms8010003 31861355 PMC7022904

[41] Csörgő B, León LM, Chau-Ly IJ, Vasquez-Rifo A, Berry JD, Mahendra C, Crawford ED, Lewis JD, Bondy-Denomy J. 2020. A compact Cascade–Cas3 system for targeted genome engineering. Nat Methods 17:1183–1190. doi:10.1038/s41592-020-00980-w 33077967 PMC7611934

[42] Larrimore KE, Rancati G. 2019. The conditional nature of gene essentiality. Curr Opin Genet Dev 58–59:55–61. doi:10.1016/j.gde.2019.07.015 31470233

[43] Hashimoto M, Ichimura T, Mizoguchi H, Tanaka K, Fujimitsu K, Keyamura K, Ote T, Yamakawa T, Yamazaki Y, Mori H, Katayama T, Kato J. 2005. Cell size and nucleoid organization of engineered Escherichia coli cells with a reduced genome. Mol Microbiol 55:137–149. doi:10.1111/j.1365-2958.2004.04386.x 15612923

[44] Fei F, diCenzo GC, Bowdish DME, McCarry BE, Finan TM. 2016. Effects of synthetic large-scale genome reduction on metabolism and metabolic preferences in a nutritionally complex environment. Metabolomics 12:1–14. doi:10.1007/s11306-015-0928-y

[45] Blomfield IC, Vaughn V, Rest RF, Eisenstein BI. 1991. Allelic exchange in Escherichia coli using the Bacillus subtilis sacB gene and a temperature‐sensitive Psc101 replicon. Mol Microbiol 5:1447–1457. doi:10.1111/j.1365-2958.1991.tb00791.x 1686293

[46] Logue C-A, Peak IRA, Beacham IR. 2009. Facile construction of unmarked deletion mutants in Burkholderia pseudomallei using sacB counter-selection in sucrose-resistant and sucrose-sensitive isolates. J Microbiol Methods 76:320–323. doi:10.1016/j.mimet.2008.12.007 19150470

[47] Kunst F, Rapoport G. 1995. Salt stress is an environmental signal affecting degradative enzyme synthesis in Bacillus subtilis. J Bacteriol 177:2403–2407. doi:10.1128/jb.177.9.2403-2407.1995 7730271 PMC176898

[48] Baumann P, Baumann L, Mandel M. 1971. Taxonomy of Marine Bacteria: the Genus Beneckea. J Bacteriol 107:268–294. doi:10.1128/jb.107.1.268-294.1971 4935323 PMC246914

[49] Webb CD, Payne WJ. 1971. Influence of Na^+^ on synthesis of macromolecules by a marine bacterium. Appl Microbiol 21:1080–1088. doi:10.1128/am.21.6.1080-1088.1971 4327612 PMC377348

