# Supplementary material for: Expanding the capabilities of MuGENT for large-scale genetic engineering of the fastest-replicating species, Vibrio natriegens
Source: Microbiol Spectr. 2024 Apr 26;12(6):e03964-23. doi: 10.1128/spectrum.03964-23 (PMC11237659; doi:10.1128/spectrum.03964-23)
Supplement: Supplemental figures and tables — Fig. S1 and S2; Tables S1 and S2. [file spectrum.03964-23-s0001.pdf]

SUPPLEMENTARY DATA

A.

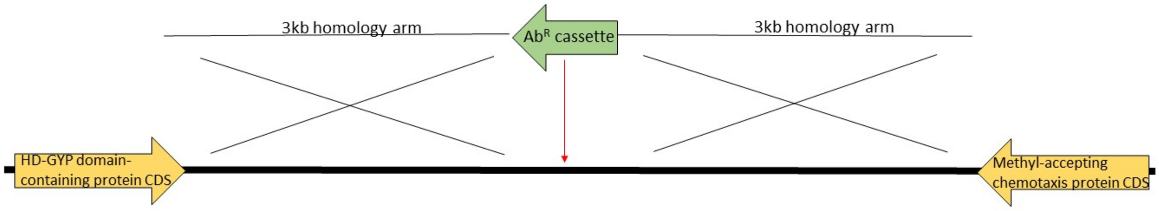

B.

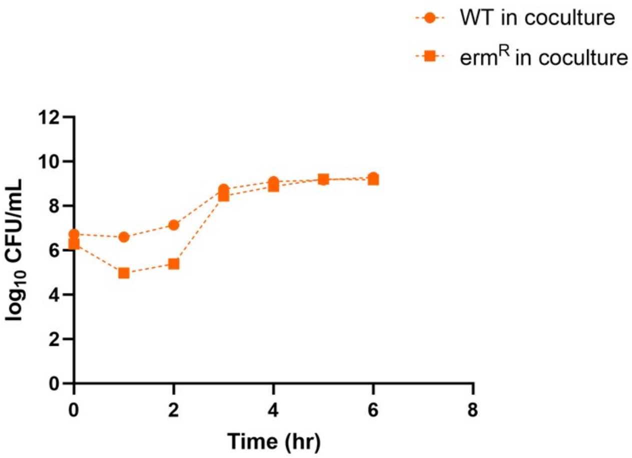

Figure S1. Antibiotic resistance gene insertion into an intergenic region results in growth defect. A) Intergenic location for antibiotic resistance gene insertion. B) Growth curves of WT and erm^R resistant marked strains of *Vibrio natriegens* grown together in coculture. Performed in duplicate biological replicates, in LB3 media at 37°C.

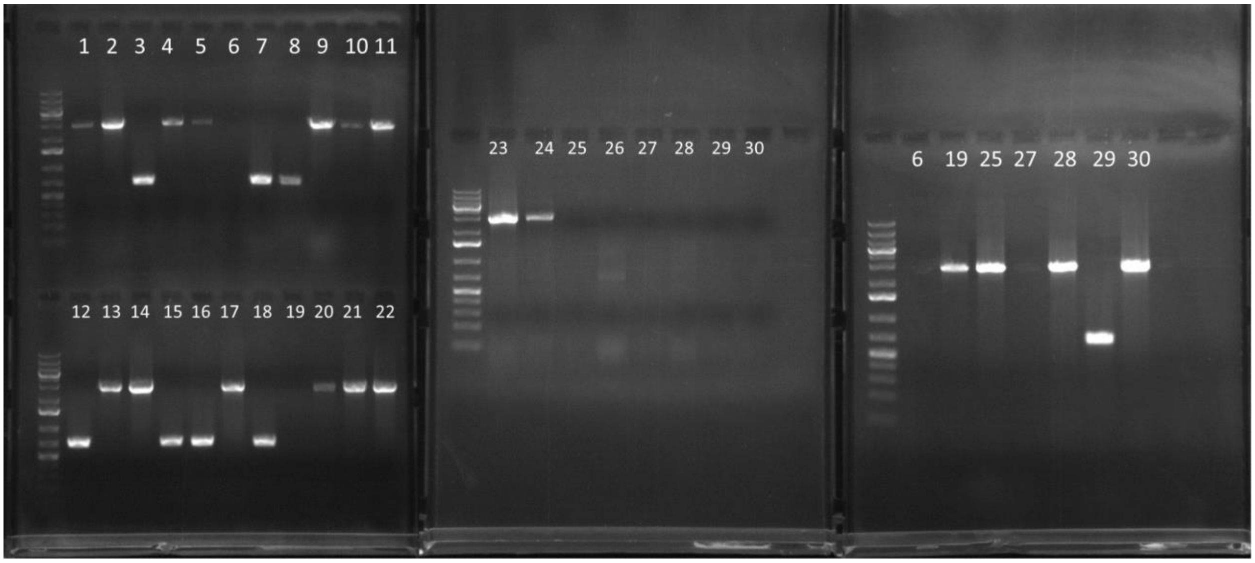

Figure S2. Colony PCR for single gene deletion efficiency analysis. Colony PCR was performed on individual colonies obtained from transformation with a construct targeting deletion of a single gene, *katG,* ~2kb in size. A band of ~3kb indicates no deletion occurred, while a band of ~800kb indicates that the deletion did occur. Numbers represent the colony isolate # ran in each column. The far right gel shows re-runs of failed reactions from the first two gels. The Thermofisher™ GeneRuler 1kb Plus Ladder was used in all gels.

Name	Description	Source
Vibrio natriegens ATCC 14048 SAD1302	WT	Ankur Dalia
Vibrio natriegens ATCC 14048 pMMB*tfox* TND1964	Amp^R	Ankur Dalia
E.coli WM3064	*thrB1004 pro thi rpsL hsdS lacZDM15 RP4-1360 Δ(araBAD)567 ΔdapA1341::[erm pir],* pir^+	[16]
pMMB*sacBtfoX*	Carries kan^R, $sucrose^s$	Kim Orth [12]
Vibrio natriegens ATCC 14048 pMMB*tfox* erm^R (EZ262)	Chr.1 Δ1,520,008-1520031::erm^R, Amp^R	This study
Vibrio natriegens ATCC 14048 pMMB*tfox* $spec^R$ (EZ263)	Chr.1 Δ1,520,008-1520031::$spec^R$, Amp^R	This study
Vibrio natriegens ATCC 14048 pMMB*sacBtfoX* (EZ276)	Kan^R,	This study
Vibrio natriegens ATCC 14048 (EZ278)	Chr.1 Δ1,520,008-1520031::$spec^R$	This study
Vibrio natriegens ATCC 14048 pMMB*tfox* Δ*oxyR* (EZ274)	Chr.1 Δ1,520,008-1520031::erm^R, Amp^R, ΔPN96_RS15030 [chr.1 Δ3,218,138 – 3,219,076]	This study
Vibrio natriegens ATCC 14048 pMMB*tfox* Δ*oxyR* Δ*dns*::*oxyR* (EZ292)	Chr.1 Δ1,520,008-1520031::$spec^R$, Amp^R, ΔPN96_RS15030 [chr.1 Δ3,218,138 – 3,219,076],	This study

	ΔPN96_RS00885 [chr.1 171,347-172,168] :: ΔPN96_RS15030 [chr.1 3,218,016 – 3,219,099]	
Vibrio natriegens ATCC 14048 pMMB*sacBtfoX* Δ280kb (EZ284)	Δ BA890_RS18565 – BA890_RS19750 [chr.2 Δ841,961 – 1,122,402],<br><br>Chr.1 Δ1,520,008-1520031::erm^R,<br><br>Kan^R	This study
Vibrio natriegens ATCC 14048 Δ280kb (EZ289)	Δ BA890_RS18565 – BA890_RS19750 [chr.2 Δ841,961 – 1,122,402],<br><br>Chr.1 Δ1,520,008-1520031::erm^R	This study

Table S1. Strains and plasmids used in this study.

Primer name	Primer sequence 5'->3'	Description	Template DNA
IGRF1	TCGTGAGGAAGGTAGTGTGT	Upstream arm of intergenic site, forward primer	*V. natriegens* ATCC14048, NZ_CP009977
IGRR1	<u>ACACAATCGCTCAAGACGTG</u>CGCCTTAGGGGTCGACTTAC	Upstream arm of intergenic site, reverse primer	*V. natriegens* ATCC14048, NZ_CP009977
IGRF2	<u>CTAATTCCCATGTCAGCCGT</u>CCGAAAGGCGTAGTCGATGG	Downstream arm of intergenic	*V. natriegens* ATCC14048, NZ_CP009977

		site, forward primer	
IGRR2	CGCCCAACACACACCACATA	Downstream arm of intergenic site, reverse primer	*V. natriegens* ATCC14048, NZ_CP009977
PF1	CACAGTGAGGACAGATTAAACGA	Upstream arm of phage region, forward primer	*V. natriegens* ATCC14048, NZ_CP009977
PR1	ACACAATCGCTCAAGACGTGACGATTACGTATCTCTGATGCGA	Upstream arm of phage region, reverse primer	*V. natriegens* ATCC14048, NZ_CP009977
PF2	CTAATTCCCATGTCAGCCGTCCAAAGGGTCACCACCGTAA	Downstream arm of phage region, forward primer	*V. natriegens* ATCC14048, NZ_CP009977
PR2	GTGACAGAGACGCAGGACTC	Downstream arm of phage region, reverse primer	*V. natriegens* ATCC14048, NZ_CP009977
ErmFwd	CACGTCTTGAGCGATTGTGTAACTGAGGATCCGGTGATTG	Erm^R gene forward primer	pEVS170
ErmRev	ACGGCTGACATGGGAATTAGGAAGCAAACTTAAGAGTGTGTTGA	Erm^R gene reverse primer	pEVS170

SmRFwd2	CACGTCTTGAGCGATTGTGTATGACCCTGCTGATTGGTTC	Spec^R gene forward primer	pAM5057
SmRRev2	ACGGCTGACATGGGAATTAGGATGTTATGGAGCAGCAACG	Spec^R gene reverse primer	pAM5057
pKD4KanF	CACGTCTTGAGCGATTGTGT	Ab^R scar forward primer	
pKD4KanR	ACGGCTGACATGGGAATTAG	Ab^R scar reverse primer	
OxyRF1	GCCGTAACGTTGGCCTATAA	Upstream arm of Δ*oxyR,* forward primer	*V. natriegens* ATCC14048, NZ_CP009977
OxyRR1	GCTAATTCAGTTTAAGCGGCCATCACACGATTCGCTTCTATTTAGTG	Upstream arm of Δ*oxyR,* reverse primer	*V. natriegens* ATCC14048, NZ_CP009977
OxyRF2	ATGGCCGCTTAAACTGAATTAGCAGCTATTGGCTCCCCTTGTT	Downstream arm of Δ*oxyR,* forward primer	*V. natriegens* ATCC14048, NZ_CP009977
OxyRR2	CACGACCGCGAACTCTATTT	Downstream arm of Δ*oxyR,* reverse primer	*V. natriegens* ATCC14048, NZ_CP009977
3217813F	GAGGCAAATGGCTTGAAGAA	Δ*oxyR* scar, forward primer	*V. natriegens* ATCC14048,

			NZ_CP009977
3219406R	CCTTGGTCAAGGCCAAGTAT	Δ*oxyR* scar, reverse primer	*V. natriegens* ATCC14048, NZ_CP009977
endAF1	CTAACATGGCTAAGCACCTG	Upstream arm of Δ*dns,* forward primer	*V. natriegens* ATCC14048, NZ_CP016345
endAR1	<u>ACACAATCGCTCAAGACGTG</u>ACTGAGGATTAGGAAAGCTGGA	Upstream arm of Δ*dns,* reverse primer	*V. natriegens* ATCC14048, NZ_CP016345
endAF2	<u>ATGGCCGCTTAAACTGAATTAGC</u>CCTCACCAATCGCGACAATC	Downstream arm of Δ*dns,* forward primer	*V. natriegens* ATCC14048, NZ_CP016345
endAR2	TAAGGTGTCTCAAATCTCAATCTAGG	Downstream arm of Δ*dns,* reverse primer	*V. natriegens* ATCC14048, NZ_CP016345
oxyRcompF1	<u>CACGTCTTGAGCGATTGTGT</u>TCGGCTCGTGTCTGTTCTG	*oxyR* complement, forward primer	*V. natriegens* ATCC14048, NZ_CP009977
oxyRcompF2	<u>GCTAATTCAGTTTAAGCGGCCAT</u>AACAACAAGGGGAGCCAATAG	*oxyR* complement, reverse primer	*V. natriegens* ATCC14048, NZ_CP009977
2728836F	TTCCCTATTCCCAGCCTGAC	Δ*dns*::*oxyR* scar, forward primer	*V. natriegens* ATCC14048, NZ_CP016345

2730429R	CTACGCGCTCAGAGATGTCT	Δ*dns*::*oxyR* scar, reverse primer	*V. natriegens* ATCC14048, NZ_CP016345
838941F	GCTGGCACATTTTACGCATAG	Upstream arm of Δ280, forward primer	*V. natriegens* ATCC14048, NZ_CP016346
841961R	GCTAATTCAGTTTAAGCGGCCATTAAGAAAAACGCTGCCCTTG	Upstream arm of Δ280, reverse primer	*V. natriegens* ATCC14048, NZ_CP016346
KatG3F2	ATGGCCGCTTAAACTGAATTAGCTCGCTAAGCTAGCATAAGCTCT	Downstream arm of Δ280, forward primer	*V. natriegens* ATCC14048, NZ_CP016346
KatG3R2	GGATTCTGACTGGAGCAAGC	Downstream arm of Δ280, reverse primer	*V. natriegens* ATCC14048, NZ_CP016346
840291F	CGGCTAATCTGACCATGAAC	Δ280 scar, forward primer	*V. natriegens* ATCC14048, NZ_CP016346
1122735R	GTTCCGGAGATATCGGACAA	Δ280 scar, reverse primer	*V. natriegens* ATCC14048, NZ_CP016346
KatG3F1	ATAGCCACCATCGGTTGAGA	Upstream arm of Δ*katG*, forward primer	*V. natriegens* ATCC14048, NZ_CP016346
KatG3R1	GCTAATTCAGTTTAAGCGGCCATCTGGCTTTTTGCGTATCCAT	Upstream arm of Δ*katG*,	*V. natriegens* ATCC14048,

		reverse primer	NZ_CP016346
KatG3F2	ATGGCCGCTTAAACTGAATTAGCTCGCTAAGCTAGCATAAGCTCT	Downstream arm of Δ*katG*, forward primer	*V. natriegens* ATCC14048, NZ_CP016346
KatG3R2	GGATTCTGACTGGAGCAAGC	Downstream arm of Δ*katG*, reverse primer	*V. natriegens* ATCC14048, NZ_CP016346
1119752F	GCCAGACATCCTAATGCCTTT	Δ*katG* scar, forward primer	*V. natriegens* ATCC14048, NZ_CP016346
1122735R	GTTCCGGAGATATCGGACAA	Δ*katG* scar, reverse primer	*V. natriegens* ATCC14048, NZ_CP016346
pMMBOriVF	AACCTGCAAACCCAGCAG	pMMB*sacBtfoX* origin forward primer	pMMB*sacBtfoX*
pMMBOriVR	CCGCTAAAGCGGCTAAAAG	pMMB*sacBtfoX* origin reverse primer	pMMB*sacBtfoX*

Table S2. List of primers used in this study. Underlined nucleotides specify homologous regions added for SOE PCR.
